# The potential of phosphorylated α‐synuclein as a biomarker for the diagnosis and monitoring of multiple system atrophy

**DOI:** 10.1111/cns.14678

**Published:** 2024-04-04

**Authors:** Toufik Abdul‐Rahman, Ranferi Eduardo Herrera‐Calderón, Arjun Ahluwalia, Andrew Awuah Wireko, Tomas Ferreira, Joecelyn Kirani Tan, Maximillian Wolfson, Shankhaneel Ghosh, Viktoriia Horbas, Vandana Garg, Asma Perveen, Marios Papadakis, Ghulam Md Ashraf, Athanasios Alexiou

**Affiliations:** ^1^ Medical Institute Sumy State University Sumy Ukraine; ^2^ Center for Research in Health Sciences (CICSA), Faculty of Medicine Anahuac University North Campus Huixquilucan Mexico; ^3^ School of Medicine, Queen's University Belfast Belfast UK; ^4^ Department of Clinical Neurosciences, School of Clinical Medicine University of Cambridge Cambridge UK; ^5^ Faculty of Medicine University of St Andrews St Andrews Scotland UK; ^6^ Humanitas University Milano Italy; ^7^ Institute of Medical Sciences and SUM Hospital, Siksha 'O' Anusandhan Bhubaneswar India; ^8^ Department of Pharmaceutical Sciences Maharshi Dayanand University Rohtak Haryana India; ^9^ Glocal School of Life Sciences Glocal University Saharanpur Uttar Pradesh India; ^10^ Princess Dr. Najla Bint Saud Al‐Saud Center for Excellence Research in Biotechnology King Abdulaziz University Jeddah Saudi Arabia; ^11^ Department of Surgery II, University Hospital Witten‐Herdecke University of Witten‐Herdecke Wuppertal Germany; ^12^ Department of Medical Laboratory Sciences University of Sharjah, College of Health Sciences, and Research Institute for Medical and Health Sciences Sharjah UAE; ^13^ University Centre for Research & Development Chandigarh University Mohali Punjab India; ^14^ Department of Research & Development Athens Greece; ^15^ Department of Research & Development AFNP Med Wien Austria; ^16^ Department of Science and Engineering Novel Global Community Educational Foundation New South Wales Australia

**Keywords:** biomarker, diagnosis, multiple system atrophy, phosphorylated α‐synuclein

## Abstract

**Introduction:**

Multiple system atrophy (MSA) is a rapidly progressive neurodegenerative disorder characterized by the presence of glial cytoplasmic inclusions (GCIs) containing aggregated α‐synuclein (α‐Syn). Accurate diagnosis and monitoring of MSA present significant challenges, which can lead to potential misdiagnosis and inappropriate treatment. Biomarkers play a crucial role in improving the accuracy of MSA diagnosis, and phosphorylated α‐synuclein (p‐syn) has emerged as a promising biomarker for aiding in diagnosis and disease monitoring.

**Methods:**

A literature search was conducted on PubMed, Scopus, and Google Scholar using specific keywords and MeSH terms without imposing a time limit. Inclusion criteria comprised various study designs including experimental studies, case‐control studies, and cohort studies published only in English, while conference abstracts and unpublished sources were excluded.

**Results:**

Increased levels of p‐syn have been observed in various samples from MSA patients, such as red blood cells, cerebrospinal fluid, oral mucosal cells, skin, and colon biopsies, highlighting their diagnostic potential. The α‐Syn RT‐QuIC assay has shown sensitivity in diagnosing MSA and tracking its progression. Meta‐analyses and multicenter investigations have confirmed the diagnostic value of p‐syn in cerebrospinal fluid, demonstrating high specificity and sensitivity in distinguishing MSA from other neurodegenerative diseases. Moreover, combining p‐syn with other biomarkers has further improved the diagnostic accuracy of MSA.

**Conclusion:**

The p‐syn stands out as a promising biomarker for MSA. It is found in oligodendrocytes and shows a correlation with disease severity and progression. However, further research and validation studies are necessary to establish p‐syn as a reliable biomarker for MSA. If proven, p‐syn could significantly contribute to early diagnosis, disease monitoring, and assessing treatment response.

## Graphical abstract

The potential of phosphorylated α‐Syn (p‐syn) as a biomarker for multiple system atrophy diagnosis and monitoring, is defined by the abnormal p‐syn accumulation in the brain and peripheral tissues.

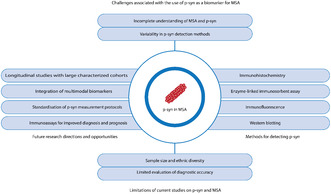

## 1 INTRODUCTION

Multiple system atrophy (MSA) is a rare, fatal neurodegenerative disorder affecting various parts of the brain, including the nigrostriatal system, cerebellum, pons, inferior olives, and key brainstem, along with the spinal cord.^1^ MSA is categorized into parkinsonian (MSA‐P) and cerebellar (MSA‐C) subtypes according to the presenting motor phenotype.^1^, ^2^ Its estimated mean incidence is 0.6–0.7 cases per 100,000 individuals, increasing to 1.6 cases per 100,000 individuals after the age of 40^3^, ^4^ with an estimated prevalence ranging from 1.9 to 3.3 cases per 100,000 individuals.^5^, ^6^

The disease is characterized by a rapid progression, leading to severe disability within 5–6 years, and typically, death within a decade of onset.^1^ MSA is predominantly considered a sporadic disease, and limited evidence suggests a genetic background.^2^, ^7^, ^8^, ^9^ Although environmental risk factors have not been consistently linked to MSA, an association with occupational exposure to certain substances has been reported.^10^, ^11^, ^12^

**FIGURE 1:**
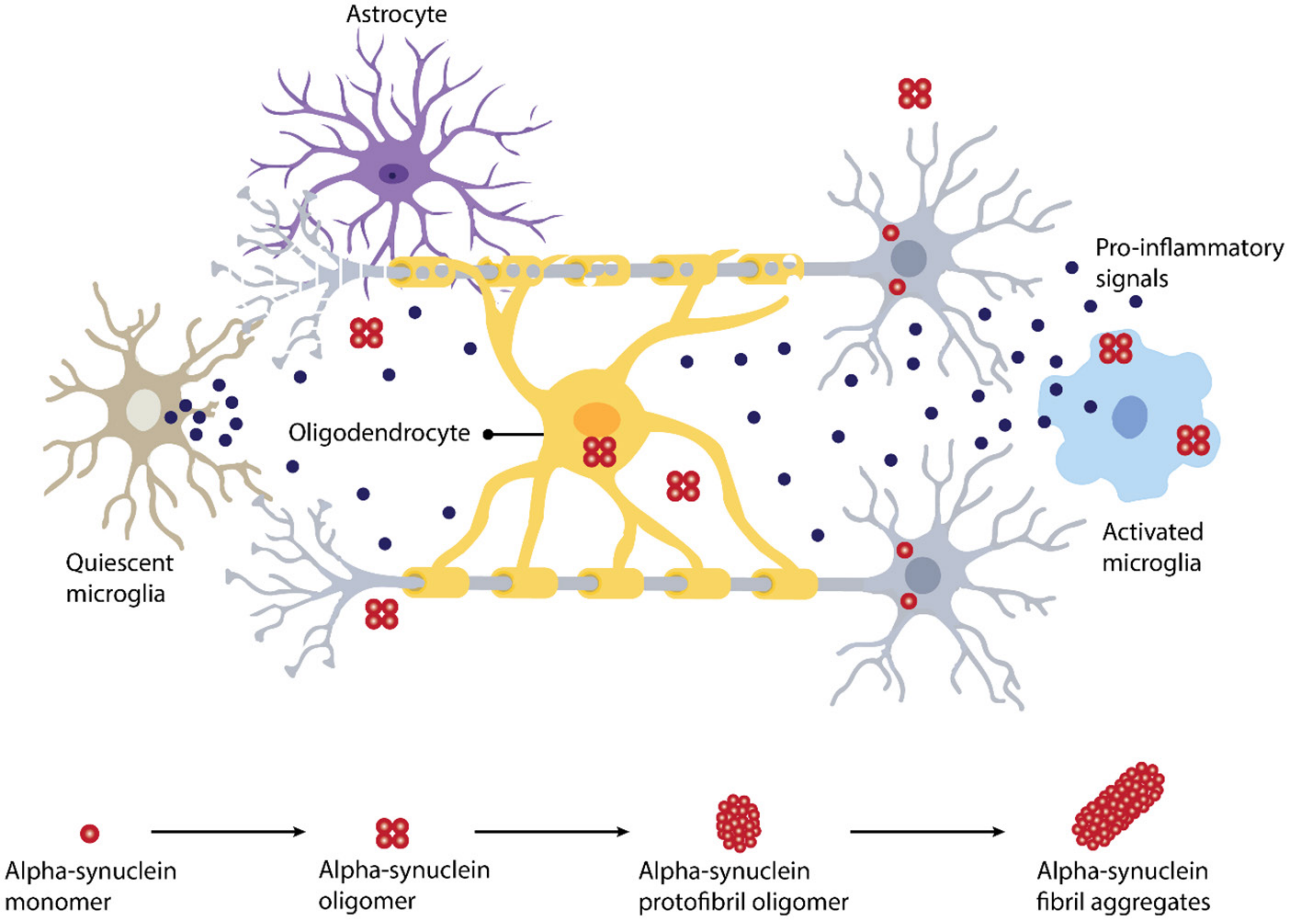
Proposed pathophysiology of MSA. MSA is characterized by misfolding and aggregation of α‐Syn in oligodendrocytes and neurons, oligodendrocyte dysbiosis, and neuroinflammation.

The pathogenic mechanisms of MSA are not fully understood, but converging evidence suggests that it is a primary oligodendrogliopathy.^13^, ^14^ MSA is characterized by the formation of glial cytoplasmic inclusions (GCIs) that disrupt neuronal support and activate microglial cells.^2^ It is a synucleinopathy marked by aggregated α‐synuclein (α‐Syn) in oligodendrocytes (Figure 1),^1^ presenting with a combination of motor and nonmotor deficits due to the variable regional distribution and severity of neuropathology.^1^, ^12^

The definitive diagnosis of MSA requires evidence of abnormal α‐Syn deposition through brain histopathology, unachievable in vivo.^15^ Differentiating early‐stage MSA from other conditions can be challenging, leading to potential misdiagnosis and subsequent problems, including incorrect treatment, distress to patients and families, and inaccurate eligibility for clinical trials.^16^ The diagnosis of MSA is supported by motor and nonmotor features, Movement of Disorder Society (MDS) criteria, and specific test findings.^17^, ^18^ Supportive clinical features, often referred to as “red flags,” include orofacial dystonia, inspiratory sighs, muscle contractures of the hands or feet, polyminimyoclonus, severe dysarthria, pathologic laughter or crying, and cold hands and feet.^17^ Autonomic dysfunction is a frequent feature of MSA, with common complaints including orthostatic hypotension, neurogenic bladder, and constipation.^19^, ^20^, ^21^ Therefore, measurements of postvoid bladder residual volume and lying/standing blood pressure are essential.^22^

Brain magnetic resonance imaging (MRI) is the gold standard imaging technique for MSA and can reveal numerous abnormalities, including atrophy of various brain regions.^23^, ^24^, ^25^, ^26^ Diffusion‐weighted imaging, magnetic resonance volumetry, and functional brain MRI are other valuable techniques for diagnosing and monitoring MSA.^16^ Additionally, brain positron emission tomography (PET) scans, SPECT scans, and cardiac sympathetic neuroimaging can help distinguish MSA from other Parkinsonian syndromes.^16^ Emerging diagnostic and monitoring methods for MSA include transcranial sonography, retinal optical coherence tomography, plasma and cerebrospinal fluid (CSF) biomarkers, and skin biopsy.^27^, ^28^, ^29^, ^30^

However, all the methods mentioned above have inherent disadvantages. Diagnosing MSA based solely on clinical symptoms is challenging, given their similarity to the clinical presentation of other more common neurodegenerative disorders. This frequently leads to misdiagnoses in MSA patients, resulting in incorrect treatment, patient and family distress, and inaccurate eligibility for clinical trials.^16^ Additionally, it is crucial to highlight that these methods primarily target a postsymptomatic approach, without accounting for a prodromal or premotor category.^31^ In a disease with high mortality rates and disease progression like this, developing a prodromal approach is essential. Since a curative treatment is unavailable, disease‐modifying therapies in the early stages become critical. Especially, when novel therapies for MSA are being developed to inhibit α‐Syn aggregation, alleviate neuroinflammation, and confer neuroprotective effects.^32^

Given the heterogeneity of the clinical presentation of MSA, the suboptimal accuracy of diagnosis, and its rapid progression, biomarkers are crucial for achieving a proper diagnosis, improving prognosis in the early stages of the disease, and monitoring patients. An ideal biomarker should be linked to the disease process, reliable, accurate, sensitive, specific, reproducible, noninvasive, acceptable to patients, and inexpensive.^33^ In the context of MSA, that biomarker could be phosphorylated α‐Syn (p‐syn), which aberrantly accumulated in both the central nervous system and peripheral tissues of MSA patients.

Several studies have identified the presence of p‐syn in the context of synucleinopathies, such as MSA.^34^, ^35^, ^36^ Notably, fibrillar p‐syn accumulation in Remak nonmyelinating Schwann cells (RSCs) has emerged as a potential specific and sensitive biomarker for MSA.^37^ Recent research has also unveiled the diagnostic potential of phosphorylated serine residues on the α‐Syn protein (pS‐α‐syn) in red blood cells (RBCs) as an indicator of MSA, suggesting its viability for early diagnosis.^38^ Furthermore, the examination of α‐Syn levels in oral mucosal cells has shown elevated results in MSA patients.^15^ Such findings contribute to the expanding body of knowledge around p‐syn as a biomarker. In addition to these developments, studies have indicated that skin biopsy measurements of p‐syn can accurately differentiate between patients with MSA and those with Parkinson's disease (PD), offering a potentially precise diagnostic tool for various synucleinopathies.^39^, ^40^

Moreover, although standardized neuroimaging, autonomic, or genetic tests may help distinguish MSA from other conditions; these tests may not be sufficient in all cases.^41^ Recent studies suggest that a combination of imaging and/or fluid biomarkers can aid in the differential diagnosis between MSA and other Parkinsonian disorders.^28^, ^42^, ^43^ CSF biomarkers, including α‐Syn, Aβ42, t‐tau, p‐tau, and NfL, are promising in diagnosing MSA.^44^

Despite the growing body of evidence strongly supporting the role of p‐syn as a valuable biomarker for MSA, a comprehensive analysis that incorporates a wide range of data sources and explores various examination methods for p‐syn as a biomarker is still lacking. With this in mind, the primary goal of this review is to provide a detailed evaluation of p‐syn's potential as a diagnostic and monitoring biomarker for MSA and encourage further research in this critical area of neurodegenerative diseases.

## 2 ALPHA‐SYNUCLEIN AND MSA

### 2.1 Alpha‐Synuclein's role in MSA

α‐Syn is a small acidic protein, typically found in presynaptic neuronal terminals, with functions in vesicular transport, membrane interactions, and neuronal plasticity.^45^, ^46^, ^47^, ^48^ MSA results from the accumulation of α‐Syn, with specific regions being more severely affected, causing damage to oligodendroglial cells and forming oligodendroglial GCIs.^49^ While MSA lacks a clearly established genetic basis, studies have identified homozygous and compound heterozygous mutations in COQ2 as potential causes of familial and sporadic MSA.^9^, ^40^ Research suggests that the pathogenesis of MSA involves various cellular processes, including oxidative stress, inflammation, microglial activation, and astrogliosis.^8^ Abnormal processing, misfolding, and aggregation of α‐Syn play a crucial role in synucleinopathies.^50^ The spread of α‐Syn pathology has been hypothesized to occur in a prion‐like manner due to its widespread presence in diseased synucleinopathy brains.^49^ Multiple studies have demonstrated the ability of human and synthetic α‐Syn aggregates to induce inclusion formation in cell culture and mouse models.^49^, ^51^, ^52^, ^53^, ^54^, ^55^, ^56^, ^57^, ^58^ This evidence indicates the prion‐like ability of α‐Syn to propagate within the nervous system, although there is no evidence suggesting that these aggregates are infectious or readily transmissible, as observed with classical prion diseases.^59^ Furthermore, some studies have found that different strains of α‐Syn can form during aggregation, leading to the proposal that distinct α‐Syn polymorphs may be responsible for the heterogeneity observed in synucleinopathies.^49^, ^51^, ^52^, ^60^, ^61^ Bousset et al. discovered two polymorphs of α‐Syn, both meeting the molecular criteria for identifying them as two strains of α‐Syn.^60^ However, they have different structures, levels of toxicity, and in vitro and in vivo seeding and propagation properties.^60^ Several studies have explored this phenomenon, and it is theorized that the specific structure of α‐Syn derived from inclusions in the brains of patients with MSA is especially toxic, capable of propagating to adjacent cells and inducing neurodegeneration.^62^, ^63^ Nevertheless, other studies have shown contradictory results, and more research is needed to determine whether pathological α‐Syn in MSA patients differs from that in patients with other Parkinsonian disorders.^64^

### 2.2 Role of posttranslational modifications (PTMs) on α‐Syn aggregation

The native conformation of α‐Syn can be perturbed by mutations, environmental factors, or PTMs. A myriad of PTMs affecting α‐Syn has been elucidated, encompassing acetylation, glycosylation, glycation, nitration, phosphorylation, ubiquitination, SUMOylation, and truncation.^65^ Compelling evidence substantiates the pivotal role of PTMs in modulating α‐Syn's aggregation propensity, solubility, turnover, membrane affinity, size, structure, charge, and interactions with other proteins.^66^, ^67^, ^68^, ^69^, ^70^, ^71^

Consequently, investigations into α‐Syn and posttranslationally modified α‐Syn in accessible tissues have been pursued as promising biomarkers for synucleinopathies. Among the plethora of PTMs, phosphorylation emerges as the most extensively studied in the context of synucleinopathies. Specifically, phosphorylation at serine 129 (p‐ser129) stands out as a hallmark for mature α‐Syn aggregates, with elevated levels discerned in the CSF and plasma of PD patients, exhibiting a correlation with symptom severity.^72^, ^73^, ^74^ In normal brains, only a modest 4% of α‐Syn is phosphorylated, whereas in synucleinopathies, notably at serine residues 129 and 87 (S129‐P, S87‐P), approximately 90% is phosphorylated within Lewy bodies (LBs).^75^, ^76^ Given the growing evidence advocating the potential of p‐syn as a biomarker of MSA, this review will focus on these specific PTMs. Nevertheless, it is imperative to underscore the necessity for further research elucidating the role and potential of other PTMs in the context of MSA.

### 2.3 Differences between p‐syn and non‐p‐syn in MSA

Both non‐p‐syn and p‐syn are involved in MSA, but p‐syn is the pathological form.^38^, ^77^ The main difference between these two forms is the presence or absence of phosphate groups on specific amino acid residues of the protein.^78^ Studies have shown that p‐syn is more prone to aggregation and is found in higher levels in MSA brain tissue compared to non‐p‐syn.^28^, ^39^ Recent studies have positioned p‐syn as one of the most promising biomarkers for MSA, while non‐p‐syn has shown inconsistent results.^39^ These differences could be explained by the pathophysiological mechanisms in which p‐syn is involved.

### 2.4 Alpha‐synuclein as a potential biomarker for MSA

Several studies on MSA have found that molecular markers related to α‐Syn show potential as diagnostic biomarkers. Shahnawaz et al. reported that the α‐Syn Real‐Time Quaking Induced Conversion (RT‐QuIC) assay for MSA disease, also known as α‐Syn protein misfolding cyclic amplification (PMCA), has a sensitivity of 95.4% in discriminating between CSF samples from patients diagnosed with PD and samples from patients with MSA.^79^, ^80^, ^81^

α‐Syn PMCA, an adaptation of the PMCA technology, has been reported as a fast and reproducible system that could be used as a high‐throughput screening method for finding new α‐Syn anti‐aggregating compounds.^82^, ^83^, ^84^, ^85^, ^86^ This assay consists of the seeding‐nucleation mechanism to cyclically amplify the process of protein misfolding.^79^ The reaction is initiated by the biological sample (seed), where the pathological α‐Syn aggregates induce the aggregation of the recombinant (rec) α ‐Syn (substrate).^87^ The kinetics of α‐Syn aggregation are monitored in real‐time by the fluorescence of thioflavin T (ThT), a dye that associates with amyloid‐β structures of the aggregating α‐Syn.^79^, ^87^

More recently, Poggiolini et al. reported that α‐Syn PMCA has a sensitivity of 75% for MSA and the potential as a monitoring method for disease progression and differentiating between synucleinopathies.^87^ Thus, due to the longer T50 of PD CSF samples but significantly lower Vmax when compared to those of MSA patients.^87^ In another study using the enzyme‐linked immunosorbent assay (ELISA), Li et al. found that p‐syn in RBCs is significantly higher in MSA patients than in controls, suggesting it could be a diagnostic marker with higher sensitivity and specificity than the plasma and CSF analysis.^38^, ^77^ Moreover, the membrane‐bound fraction of erythrocyte α‐Syn has also been found to be elevated in MSA patients, which could serve as a diagnostic biomarker.^88^ Other studies have also reported the use of CSF α‐Syn PMCA and skin biopsy samples to distinguish MSA from other neurodegenerative diseases.^79^, ^89^, ^90^ On the other hand, the quantification of total α‐Syn levels in CSF and plasma in synucleinopathies has yielded inconsistent results, with most studies finding decreased levels in MSA compared to controls.^28^, ^91^, ^92^, ^93^, ^94^

## 3 RELATIONSHIP BETWEEN P‐SYN AND MSA

Neurodegenerative diseases such as MSA and PD frequently exhibit protein aggregation. Specifically, p‐syn, the pathological form of α‐Syn, plays a vital role in MSA.^38^ Its aggregation in oligodendrocytes is an essential pathological characteristic of MSA.^95^, ^96^ Beyond being a sign of MSA, existing evidence supports the notion that mutations in the α‐Syn gene may increase the risk of MSA development. α‐Syn, a component of oligodendroglial inclusions in MSA, contains p‐syn, suggesting that α‐Syn gene mutations may lead to increased production and aggregation, contributing to MSA's pathogenesis.^97^

### 3.1 Evidence supporting p‐syn as a potential biomarker for MSA

Diagnosing MSA is challenging, owing to symptomatic similarities with other neurodegenerative disorders. While no validated biomarker exists for MSA, p‐syn exhibits potential as a diagnostic tool.^98^ Notably, MSA patients showed significantly elevated levels of p‐syn in CSF in comparison to PD patients and healthy controls.^96^ In the same vein, p‐syn levels in RBCs were significantly higher in MSA patients.^38^ These elevated levels demonstrate a high sensitivity (80%) and specificity (89%) in distinguishing MSA from healthy controls, further establishing p‐syn's potential as a diagnostic and prognostic biomarker.^38^

In the same vein, a study published in Neurology unveiled a noteworthy discovery concerning the detection of p‐syn in neurodegenerative diseases. This research found that p‐syn was predominantly located in autonomic fibers in conditions such as PD, dementia with Lewy bodies (DLB), and pure autonomic failure. Intriguingly, in the case of MSA, p‐syn was detected in somatic fibers of the upper dermis, revealing a unique and distinctive localization pattern that could offer valuable diagnostic insights.^99^

Furthermore, a study on a Chinese cohort comprising 107 MSA patients and 220 healthy controls revealed significant findings. It identified elevated levels of serine 129‐phosphorylated α‐syn (pS‐α‐syn), a primary pathological form of α‐Syn, within MSA patients' RBCs. Notably, the levels of pS‐α‐syn in RBCs were measured at 14.02 ± 4.02 ng/mg in MSA patients compared to 11.89 ± 3.57 ng/mg in healthy controls.^38^ This strongly suggested that pS‐α‐syn in RBCs could be a promising diagnostic biomarker for MSA.

In the context of skin biopsies, the presence of p‐syn has been identified in patients with Long‐COVID Postural Orthostatic Tachycardia Syndrome (POTS), even in cases where prodromal symptoms of neurodegenerative diseases are absent.^100^ This compelling revelation hints at the potential role of p‐syn as an early harbinger of neurodegenerative conditions within specific patient populations, thereby underscoring its significance in early disease detection.

Recent research has also investigated p‐syn's utility in differentiating MSA from other neurodegenerative conditions. The presence of p‐syn in skin biopsies has exhibited high sensitivity (>88%) and specificity (>85%) in diagnosing MSA, thereby facilitating its distinction from PD.^36^ Moreover, MSA patients were found to have significantly elevated p‐syn levels in CSF compared to those with progressive supranuclear palsy (PSP) or corticobasal syndrome (CBS). Such findings bolster the proposition of p‐syn as a promising biomarker to distinguish MSA from other neurodegenerative diseases.

### 3.2 Comparison of p‐syn with other potential biomarkers for MSA

The early detection of MSA is critical, as it can substantially improve patient outcomes. Various biomarkers, such as p‐syn, α‐Syn, neurofilament light chain (NFL), DJ‐1, glial fibrillary acidic protein (GFAP), and microRNAs (miRNAs), have been investigated for MSA. NFL, indicative of axonal damage and neurodegeneration, has been found to have elevated levels in the CSF of MSA patients.^101^ Further, studies such as Bridel et al. (2019) reveal that serum NFL levels can distinguish MSA from PD with high accuracy.^102^ DJ‐1, involved in cellular oxidative stress response and transcription regulation, is unique in showing decreased serum levels within the CSF of MSA patients compared to controls and PD patients, but its validity as a biomarker for MSA needs further exploration.^101^

Bridel et al. (2019) demonstrated elevated GFAP levels in the CSF of MSA patients relative to healthy and Parkinson's patients.^102^ GFAP, known as a marker of astroglial activation, has been used in the investigation of various neurodegenerative disorders and has recently emerged as a potential MSA biomarker. While proteins comprise the majority of potential MSA biomarkers, some research has also explored miRNAs as diagnostic markers. Vallelunga et al. (2021) reported considerably lower miR‐96‐5p levels in the CSF of MSA patients versus healthy controls.^103^ Additionally, a study by Bougea et al. (2022) found reduced plasma miR‐19b‐3p levels in MSA patients compared to healthy individuals.^104^ These findings are indicative of the prospective utility of miRNAs in MSA diagnosis, though at present, p‐syn and NFL seem to be the most promising.

Interestingly, the diagnostic accuracy for MSA was found to be increased by the combined assessment of p‐syn and t‐tau protein in CSF, rather than evaluating each biomarker individually.^93^

## 4 METHODS FOR DETECTION OF P‐SYN

### 4.1 Current methods for detecting p‐syn

Presently, several methods are employed to detect p‐syn (Table 1), with one of the most prevalent being immunohistochemistry (IHC). IHC uses specific antibodies to identify p‐syn in brain tissue samples from individuals diagnosed with MSA. Notably, studies by Cykowski et al. (2015) and Shults et al. (2005) have used this technique to pinpoint p‐syn in brainstem and cerebellar tissue samples from MSA patients.^105^, ^106^ Another investigation leveraged this method to discern MSA by observing LBs and the patterns of α‐Syn deposition.

**TABLE 1:** provides a comprehensive overview of the various techniques employed in the detection of p‐syn, including a concise description of the methodology as well as the advantages and disadvantages associated with each approach.

Methods for detection of P‐SYN			
Technique	Methodology	Advantages	Disadvantages
IHC	Antibody–antigen binding performed on tissue sections	‐ Highly effective	‐ Requires high‐quality brain tissue samples
WB	Antibody–antigen binding through gel electrophoresis	‐ High specificity and sensitivity	‐ Requires high‐quality brain tissue samples
IF	Antibody–antigen binding with fluorescent dyes	‐ High specificity and sensitivity	‐ Requires high‐quality brain tissue samples
ELISA	Antibody–antigen binding with enzyme‐linked secondary antibody	‐ Less invasive method	‐ Lacks specificity
		‐ Precise quantitative measurements	
MS	Measurement of the mass‐to‐charge ratio (m/z) of ions	‐ High specificity and sensitivity	‐ Vulnerable to matrix effects and other technical issues
		‐ Precise quantitative measurements	
		‐ Can detect isoforms and posttranslational alterations	
PMCA	Amplification and detection of small amounts of misfolded proteins	‐ High sensitivity	‐ Reduced specificity
		‐ Potential for early‐stage detection of the pathology	‐ Lack of standardization
PET	Imaging technique through a radiotracer with positron‐emitting radionuclide.	‐ Only noninvasive method.	‐ The accuracy and specificity of data can be affected by background signals and nonspecific binding.
		‐ Potential for early‐stage detection of the pathology	

Abbreviations: ELISA, enzyme‐linked immunosorbent assay; IF, immunofluorescence; IHC, immunohistochemistry; MS, mass spectrometry; PET, positron emission tomography; PMCA, protein misfolding cyclic amplification; WB, Western blotting.

Other approaches for p‐syn detection include Western blotting (WB) and immunofluorescence (IF).^38^, ^99^ WB, known for its ability to detect and quantify specific proteins like p‐syn, operates by employing antibodies specific to the protein in question.^38^ In parallel, IF uses antibodies in conjunction with fluorescent dyes to identify specific proteins within samples.^99^

Diverging from brain tissue samples, ELISA uses tailored antibodies to detect p‐syn in CSF samples from MSA patients. As reported by Mollenhauer et al. (2019), ELISA has proven invaluable in measuring p‐syn in MSA patients alongside other neurodegenerative disorders.^107^ Similarly employing fluid samples, methods like mass spectrometry and PMCA have been applied. Marques et al. (2021) executed the mass spectrometry approach to isolate a specific p‐syn peptide increased in the CSF of MSA patients,^108^ whereas Fairfoul et al. (2016) used PMCA to detect p‐syn in the CSF samples by amplifying and identifying small quantities of p‐syn.^81^

Beyond methods requiring samples, PET imaging presents a noninvasive alternative. Used by Christine et al. (2020), this technique involves detecting the accumulation of p‐syn within the brains of MSA patients, thereby contributing to the understanding of the presence of p‐syn in MSA as well as other neurodegenerative diseases.^109^

### 4.2 Advantages and disadvantages of the current methods

At present, the most pronounced disadvantage affecting many current methods for detecting p‐syn in patients with MSA lies in the substantial cost and the labor‐intensive time requisite to execute these techniques. IHC is a particularly effective method, enabling the detection of p‐syn in brain tissue and facilitating a comprehensive examination of its distribution and protein co‐localization. Furthermore, it allows the identification of various phosphorylated alpha‐synuclein epitopes, which could provide insights into the disease's severity and progression.^106^ However, IHC has its limitations, requiring high‐quality brain tissue samples that may not always be obtainable or feasible, particularly in living patients. Additionally, there is the risk that the tissue sample may undergo damage during processing and fixing, potentially altering epitope recognition and signal intensity.

Enzyme‐linked immunosorbent assay serves as a less invasive alternative, relying on CSF samples procured through lumbar puncture, as opposed to brain tissue samples. It can also provide high‐throughput analysis when standardized and automated, combined with a quantitative evaluation of p‐syn levels, critical for monitoring the disease's progression and thereby informing patient treatment. However, ELISA is not without drawbacks; its quantitative nature contrasts with a lack of specificity.^107^ Moreover, factors such as sample handling and storage may influence accuracy and reproducibility, leading to possible false positives and negatives.

In contrast, mass spectrometry demonstrates high sensitivity and specificity in detecting and quantifying p‐syn in CSF.^108^ This method has the ability to identify p‐syn isoforms and PTMs, potentially revealing more about the disease pathology. However, it is susceptible to matrix effects and other technical challenges that might compromise the precision and repeatability of results.

Among the techniques, PET imaging is unique as a noninvasive method. It furnishes in vivo visualization of p‐syn accumulation within the brain, facilitating early‐stage pathology detection, possibly even before symptoms manifest, and permits ongoing monitoring of disease progression.^109^ Nevertheless, PET imaging has its own set of challenges. The accuracy and specificity of the data may be undermined by background signals and nonspecific binding, tempering its overall effectiveness.

### 4.3 Future direction for improved detection methods

The detection and monitoring of MSA have witnessed significant advancements, particularly in the use of p‐syn as a diagnostic biomarker. Nevertheless, the pursuit of a faster and more precise diagnostic method continues to be actively investigated. The integration of multiple biomarkers, demonstrated by the potential synergy of combining p‐syn with other markers such as NFL and tau,^110^ shows promising potential. This multimarker approach could increase diagnostic accuracy while providing greater insight into the pathophysiology and progression of MSA. In this context, Mollenhauer et al. (2011) found that an integrated assessment of p‐syn and t‐tau protein in CSF exceeded the diagnostic accuracy achievable by evaluating each biomarker separately.^93^

Moreover, exploring ways to reduce the expression of the α‐Syn gene is a growing area of interest that may minimize the presence of α‐Syn protein aggregates, including p‐Syn.^111^ Consequently, this could potentially hinder the propagation of pathological p‐Syn and other aggregates triggered by characteristic seeding events in synucleinopathies.^111^

Emerging cellular techniques, such as the analysis of p‐Syn from skin samples and biopsies, represent promising directions in biomarker research. Initial findings underscore their potential, but comprehensive validation is required to confirm their effectiveness and wider applicability.^37^

Among these techniques, the analysis of p‐syn in skin samples offers significant promise for diagnosing MSA. The simplicity of conducting skin biopsies, with minimal patient discomfort, is a notable advantage.^112^ Detection of p‐syn in skin nerves is achieved through indirect immunofluorescence, although the method's sensitivity can vary depending on the thickness of the tissue sections examined.^113^ Thicker sections (50 μm) tend to yield a higher positivity rate for p‐syn compared to thinner sections (10 or 20 μm).^113^

Despite these advancements, challenges persist, with the sensitivity of p‐Syn detection showing substantial variability, potentially due to methodological distinctions among laboratories.^114^ Additionally, in synucleinopathies without autonomic failure, there may be a sporadic distribution of abnormal α‐Syn aggregates within skin nerves, complicating the detection process.^40^ Thus, further research is necessary to standardize these methodologies and enhance their reliability.

Collectively, these innovative strategies are poised to deepen our understanding of p‐Syn's role in MSA progression and treatment monitoring. This optimistic outlook bodes well for future research and clinical practice.

## 5 CLINICAL APPLICATIONS OF P‐SYN AS A BIOMARKER FOR MSA

Multiple system atrophy manifests as a neurodegenerative disease with variable clinical presentations, either featuring atrophy with predominant Parkinsonian features (MSA‐P) or with predominant cerebellar dysfunction (MSA‐C).^115^ Accurate diagnosis relies on a comprehensive medical history and a thorough neurological examination. Additional diagnostic tests serve to corroborate the diagnosis, exclude other differential diagnoses, and inform treatment strategies.^116^

According to criteria adapted from Gilman et al., a definitive diagnosis of MSA requires neuropathological evidence of GCIs positive for α‐Syn in conjunction with neurodegenerative changes in either striatonigral or olivopontocerebellar structures.^117^

Biopsy and PET are two diagnostic modalities employed for detecting α‐Syn accumulation in MSA.^118^ A study by Doppler et al. revealed that 67% of MSA patients exhibited detectable p‐syn in dermal nerve fibers, with the sensitivity increasing to 75% and 73% through the evaluation of serial sections.^119^ Interestingly, the study found that p‐syn was concentrated in unmyelinated somatosensory fibers in MSA.

Further validation for the clinical utility of p‐syn as a biomarker comes from a study by Donadio et al., which reported p‐syn aggregates in 78% of their MSA patient cohort.^37^ The study also stated that 74% of MSA patients were positive for p‐syn in skin RSCs, a finding absent in patients with PD or DLB. Subsequent immuno‐electron microscopy analyses confirmed the exclusive presence of Schwann cell cytoplasmic inclusions in MSA, and not in PD/DLB.^37^

P‐syn in CSF holds promise as a biomarker, reflecting the complex molecular dynamics of the central nervous system. It provides valuable insights into MSA pathology, thereby improving diagnostic precision and monitoring disease trajectory.^120^

In addition to its clinical diagnostic potential, p‐syn may also have therapeutic implications for MSA. Numerous investigations have assessed the effectiveness of immunotherapies targeting α‐Syn and p‐syn in pre‐clinical, animal models.^121^ Additionally, under the umbrella of passive immunization, clinical trials to investigate the efficacy and safety of PRX002, a monoclonal antibody targeting multiple forms of α‐Syn (including p‐syn) have been undertaken and are producing promising results.^122^, ^123^ Furthermore, active immunization in murine models against p‐syn has helped produce specific antibodies targeting the phospho‐Ser422 epitope and demonstrated a notable reduction in insoluble Tau species through both biochemical and immunohistochemical analyses with a promotion in Tau clearance from the brain to the periphery, as post‐immunization blood samples showed elevated Tau levels.^124^

Immunotherapy poses several challenges in treating synucleinopathies due to intracellular α‐Syn aggregation, poor blood–brain barrier permeability of antibodies, and passive or active administration of antibodies that can stimulate endogenous antibody production. Nevertheless, a multidisciplinary approach with a combination of disease‐modifying therapies and a precise patient selection process may potentially treat synucleinopathies in the future.

Despite these advances, immunotherapeutic approaches encounter challenges such as intracellular α‐Syn aggregation and poor blood–brain barrier permeability of antibodies. However, a multidisciplinary approach combining disease‐modifying therapies and precise patient selection could provide future avenues for treating synucleinopathies effectively.

## 6 CHALLENGES ASSOCIATED WITH THE USE OF P‐SYN AS A BIOMARKER FOR MSA

### 6.1 Variability in p‐syn detection methods

Numerous techniques have been employed for the detection of p‐syn, including IHC, ELISAs, and proximity ligation assays (PLAs). However, these methods often yield inconsistent results due to differences in tissue preparation, antibody specificity, and epitope recognition. These inconsistencies hinder the standardization and comparability of α‐Syn and its aggregates' (including p‐syn) measurements across studies.

In a study by Patricca et al.^125^ which aimed to assess the effectiveness of various assays in identifying the full range of α‐Syn proteoforms relevant to the disease, it was revealed that none of the three immunoassays investigated effectively detected the complete spectrum of α‐Syn species associated with the disease.

Notably, another study found that almost all MSA patients and the majority of PD patients had evidence of p‐syn in at least one skin biopsy. However, MSA patients exhibited more extensive p‐syn deposition and a wider peripheral distribution than their PD counterparts, suggesting the substantial impact of tissue preparation methods on p‐syn detection in MSA.^40^

Another investigation highlighted variations in the detection of α‐Syn pathology depending on the type of antibodies used. Vaccine‐generated antibodies were found to detect more α‐Syn pathology compared to commercially available α‐Syn antibodies. The levels of α‐Syn immunoreactivity varied among brain regions and disease types, with one antibody (IGG‐3) demonstrating high recognition levels, particularly in brain regions affected early in the disease progression. Importantly, IGG‐3 displayed a strong affinity for glial inclusions commonly found in MSA, known for their more compact conformation.^126^

Furthermore, epitope imprinting, a versatile strategy for protein recognition, offers flexibility in epitope selection. However, the selection of epitope peptide sequences and functional monomers can significantly impact the results. For instance, a technique involving a glycated C‐terminus nonapeptide epitope anchored onto a boronic acid‐functionalized substrate, followed by controlled oriented surface imprinting through the polycondensation of multiple silylating reagents, presents the potential for enhancing the reliability of epitope recognition in p‐syn detection.^127^

These factors collectively underscore the complexity of p‐syn detection in MSA and underscore the importance of precise considerations in enhancing the accuracy and reliability of diagnostic methodologies. Furthermore, These findings underscore the urgent need for the development of advanced α‐Syn immunoassays capable of encompassing the full spectrum of α‐Syn proteoforms relevant to neurodegenerative diseases.

### 6.2 Incomplete understanding of MSA and p‐syn

Several methodologies, including IHC, ELISAs, and PLAs, have been employed for the detection of p‐syn. However, these techniques are not without limitations. Factors such as tissue preparation protocols, antibody specificity, and epitope recognition can introduce variability, thereby impeding the standardization and comparability of α‐Syn measurements, including its phosphorylated forms, across different studies. For instance, a study by Patricca et al. scrutinized the effectiveness of different immunoassays in detecting the spectrum of α‐Syn proteoforms relevant to MSA.^125^ Their findings revealed that none of the three immunoassays investigated could comprehensively identify the entire array of α‐Syn species relevant to the disease. These findings emphasize the urgent need for the development of advanced, more accurate immunoassays that can capture the full range of α‐Syn proteoforms.

The identification and validation of reliable biomarkers for MSA require a multidisciplinary approach to understanding the complex pathological mechanisms underlying this neurodegenerative disorder. Improved understanding in this domain is critical for the development of early detection strategies and therapeutic interventions, particularly given the current lack of curative options for MSA.

## 7 LIMITATIONS OF CURRENT STUDIES ON P‐SYN AND MSA

### 7.1 Sample Size and Ethnic Diversity

A literature review by Magalhães et al., focusing on the utility of α‐Syn and its aggregates as biomarkers for synucleinopathies, revealed that many studies examining PTMs of α‐Syn as biomarkers have suffered from limited sample sizes.^128^ This lack of sufficient sample sizes compromises the ability to assess patient variability and the reproducibility of results across diverse laboratories. Notably, the majority of these studies have primarily concentrated on white populations of American, European, and Asian descent. There is a glaring absence of research involving underrepresented ethnic groups, such as African Americans, Africans, and Middle Eastern populations. This lack of representation underscores the imperative for more comprehensive studies that encompass a broad range of ethnicities.^128^

### 7.2 Limited evaluation of diagnostic accuracy

The rigorous evaluation of p‐syn as a diagnostic biomarker for MSA requires meticulous investigation. Current research often falls short in undertaking exhaustive evaluations of diagnostic accuracy, thereby limiting the reliability of p‐syn as a diagnostic tool. A study by Dutta et al. highlighted the rarity of MSA as a challenge in obtaining sufficient biofluid samples for analysis.^129^ Furthermore, the heterogeneous origins of samples, especially concerning MSA, have led to inconsistent clinical measures. For instance, clinics in the United States specializing in movement disorders frequently employ the Unified Multiple System Atrophy Rating Scale (UMSARS) for disease assessment, whereas clinics focused on ataxia use the Scale for the Assessment and Rating of Ataxia (SARA). This lack of uniformity impedes the comparison of datasets across different clinical settings.^129^

## 8 FUTURE RESEARCH DIRECTIONS AND OPPORTUNITIES

### 8.1 Longitudinal studies with large and well‐characterized cohorts

**FIGURE 2:**
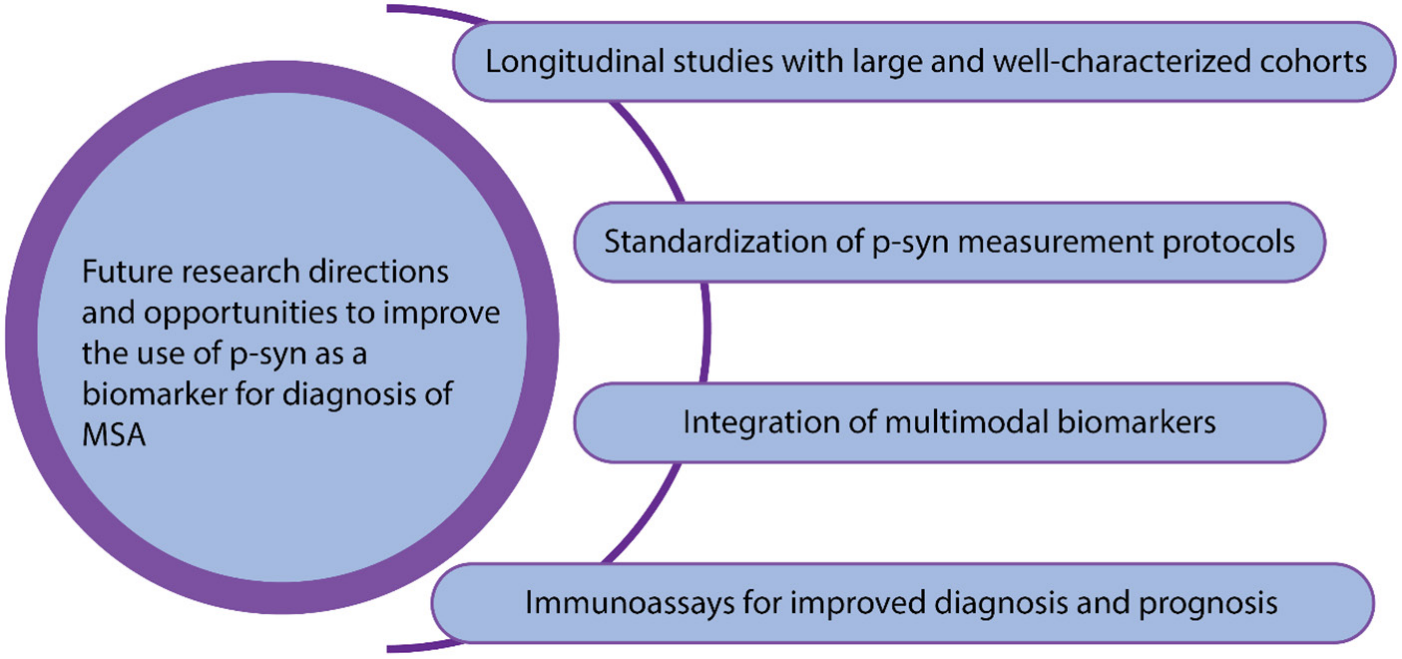
Future research directions and avenues to improve the use of p‐syn for the diagnosis of MSA. This figure depicts future research perspectives and avenues to improve the use of p‐syn for the diagnosis of MSA. These include prioritizing longitudinal studies with large and well‐characterized cohorts, standardization of p‐syn measurement protocols, integration of multimodal biomarkers, and immunoassays for improved diagnosis and prognosis.

Future endeavors should prioritize longitudinal studies incorporating large and well‐characterized MSA cohorts (Figure 2). Such an approach would not only improve statistical power but also extend the generalisability of findings. Employing repeated measures of p‐syn in combination with rigorous clinical evaluations will offer invaluable insights into the biomarker's temporal fluctuations as well as its prognostic capabilities in tracking disease evolution.

### 8.2 Standardization of p‐syn measurement protocols

The reliable measurement of p‐syn across various studies requires the development of standardized protocols. To that end, efforts should focus on the formulation of consensus guidelines, reference standards, and stringent quality control measures. These will mitigate inter‐laboratory variability, thereby increasing the reproducibility and dependability of p‐syn measurements.

### 8.3 Integration of multimodal biomarkers

The combination of p‐syn quantifications with alternative biomarkers, such as neuroimaging, CSF analytes, and relevant genetic markers, could substantially improve the diagnostic accuracy of MSA. Future research should thus explore the integration of such multimodal biomarker platforms. This approach is likely to improve diagnostic accuracy and pave the way for earlier disease detection.

### 8.4 Immunoassays for improved diagnosis and prognosis

To overcome the current limitations, advanced immunoassays are being developed to provide more accurate diagnostic and prognostic biomarkers for MSA. For instance, a study aimed to develop a unique cutaneous pathologic signature of p‐syn that could distinguish patients with MSA from patients with PD and healthy controls. The results provided >90% sensitivity and specificity in distinguishing between the two disorders.^40^ Another research initiative focused on the quantitation of alpha‐synuclein human brain proteoforms, suggesting disease‐specific biochemical profiles of synucleinopathies. The study used multiplexed and quantitative immunoassay‐based approaches in human brain extracts to point to disease‐specific biochemical alpha‐synuclein proteoform profiles in distinct neurodegenerative disorders.^130^

## 9 CONCLUSION

Multiple system atrophy is a devastating neurodegenerative disorder with limited treatment options and a rapid progression leading to severe disability and death. The complexity of establishing an accurate and early diagnosis is exacerbated by symptom overlap with other neurodegenerative conditions and a lack of specific biomarkers. P‐syn has emerged as a potential biomarker, demonstrating diagnostic and prognostic utility across a range of biological matrices, including CSF, RBCs, oral mucosal cells, and dermal tissues. Notably, CSF and RBC measurements exhibit high sensitivity and specificity in distinguishing MSA from other neurodegenerative diseases.

Nonetheless, significant challenges must be addressed for p‐syn to become a reliable diagnostic tool. Variability in detection methods and our incomplete understanding of MSA and p‐syn remain obstacles to standardizing diagnostic techniques. Additionally, addressing limitations in sample size, ethnic diversity, and diagnostic accuracy evaluation is crucial to enhance the reliability of p‐syn as a diagnostic marker.

Future research directions should include large‐scale longitudinal studies with well‐characterized cohorts, standardization of p‐syn measurement protocols, and the integration of multimodal biomarkers to improve diagnostic accuracy. Advanced immunoassays offer a promising avenue for more accurate diagnosis and prognosis of MSA. These advancements in research and clinical practice will not only enhance our understanding of MSA but also facilitate earlier disease detection, monitoring, and evaluation of disease progression, as well as more effective therapeutic interventions.

## AUTHOR CONTRIBUTIONS

Conceptualization of ideas: Toufik Abdul‐Rahman. Data curation: Toufik Abdul‐Rahman, Andrew Awuah Wireko, Tomas Ferreira. Visualization: Toufik Abdul‐Rahman. Writing of Initial Draft: Ranferi Eduardo Herrera‐Calderón, Arjun Ahluwalia, Maximillian Wolfson, Shankhaneel Ghosh. Writing Final Manuscript—Review and Editing: Toufik Abdul‐Rahman, Andrew Awuah Wireko, Tomas Ferreira, Joecelyn Kirani Tan, Viktoriia Horbas, Vandana Garg, Asma Perveen, Marios Papadakis, Ghulam Md Ashraf, and Athanasios Alexiou.

## CONFLICT OF INTEREST STATEMENT

The authors report no potential competing interests.

## FUNDING INFORMATION

The Deanship of Scientific Research (DSR) at King Abdulaziz University, Jeddah, Saudi Arabia has funded this project (grant no. KEP‐36‐130‐42). The authors, therefore, acknowledge with thanks DSR's technical and financial support.

## Data Availability

Data available within the article or its supplementary materials.
